# Multidisciplinary care for patients with tracheostomy shortened time to decannulation

**DOI:** 10.1186/cc10747

**Published:** 2012-03-20

**Authors:** A Van Hees, F Van Beers, J Van Rosmalen, D Ramnarain, W Van den Wildenberg

**Affiliations:** 1 St Elisabeth Hospital, Tilburg, the Netherlands

## Introduction

Care for cannulated patients in our hospital is not uniform and clear, leading to an unnecessarily long period of cannulation and even unsafe situations. Therefore our hospital formed a specialized multidisciplinary cannula team (SMCT) consisting of an intensivist, three ventilation practitioners and a registered nurse. The aim of this team was to shorten the period of cannulation and to guarantee uniform care and safety around cannulated patients in our ICU and on the ward.

## Methods

The study was conducted in the mixed medical and neurosurgical ICU of a teaching hospital with a duration of 22 months. Two groups of patients were studied. Group one received PDT before introduction of a SMCT (*n *= 49) and group two received PDT (*n *= 27) after introduction of a SMCT. During treatment in the ICU and after discharge, all patients were followed by the cannula team. This team eventually made the decision to decannulate the patients. We evaluated the results of a multidisciplinary cannula team (SMCT) during a follow-up period of 22 months.

## Results

For patient data see Table 1. Seventy-six patients were included in this study. The study showed a reduction in time to decannulation after a mechanical ventilation period of 8.4 days. However clinically relevant, this was not statistically significant.

**Table 1:** Results of comparative analysis between patients before and after the cannula team

	Pre-cannula team (*n *= 49)	Post-cannula team (*n *= 27)
Age (years)	52.2 ± 16.3	56.4 ± 16.8
APACHE II score	20.9 ± 5.2	21.4 ± 5.7
Intubated days before tracheostomy	12.8 ± 7.9	9.9 ± 7.3
Length of stay in ICU	34.1 ± 4.7	36.6 ± 28.9
Cannulation days	22 ± 15.4	20 ± 0.7
Mechanical ventilation after tracheostomy	9.3 ± 7.4	9.1 ± 9
Tracheostomy after mechanical ventilation	18.2 ± 27.7	9.8 ± 9.5

## Conclusion

With the introduction of a SMCT a clinically relevant reduction of cannulation period could be achieved. The group was small and probably underpowered to show a statistically significant reduction in the cannulation period.

